# Supplementary material for: Humanization of the Reaction Specificity of Mouse Alox15b Inversely Modified the Susceptibility of Corresponding Knock-In Mice in Two Different Animal Inflammation Models
Source: Int J Mol Sci. 2023 Jul 3;24(13):11034. doi: 10.3390/ijms241311034 (PMC10341735; doi:10.3390/ijms241311034)
Supplement: Supplementary file 1 [file ijms-24-11034-s001.zip › ijms-2409594-supplementary.pdf]

Supplementary Materials

Humanization of the Reaction Specificity of Mouse Alox15b Inversely Modified the Susceptibility of Corresponding Knock-In Mice in Two Different Animal Inflammation Models

Marjann Schäfer [1,2], Florian Reisch [1,2], Dominika Labuz [3], Halina Machelska [3], Sabine Stehling [1], Gerhard P. Püschel [2], Michael Rothe [4], Dagmar Heydeck [1] and Hartmut Kuhn [1,*]

[1] Department of Biochemistry, Charité—Universitätsmedizin Berlin, Corporate Member of Freie Universität Berlin and Humboldt-Universität zu Berlin, Charitéplatz 1, D-10117 Berlin, Germany;
marjann.schaefer@googlemail.com (M.S.); reisch_florian@web.de (F.R.);
sabine.stehling@charite.de (S.S.); dagmar.heydeck@charite.de (D.H.)

[2] Institute for Nutritional Sciences, University Potsdam, Arthur-Scheunert-Allee 114–116, D-14558 Nuthetal, Germany; gpuesche@uni-potsdam.de

[3] Department of Experimental Anesthesiology, Charité—Universitätsmedizin Berlin, Corporate Member of Freie Universität Berlin and Humboldt-Universität zu Berlin, Hindenburgdamm 30,
D-12203 Berlin, Germany; dominika.labuz@op.pl (D.L.); hmachelska@gmail.com (H.M.)

[4] Lipidomix GmbH, Robert-Roessle-Straße 10, D-13125 Berlin, Germany;
michael.rothe@lipidomix.de

* Correspondence: hartmut.kuehn@charite.de; Tel.: +49-30-450528040

Table S1. Detection limits for the hydroxy fatty acids analyzed in this study.

No.	Parent fatty acid	Metabolites	Detection limit (ng)
1	Docosahexaenoic acid	4-HDHA	0.13
2		7-HDHA	0.22
3		8-HDHA	0.43
4		8-HDHA	0.43
5		11-HDHA	0.14
6		13-HDHA	0.62
7		14-HDHA	0.11
8		16-HDHA	0.12
9		17-HDHA	0.38
10		20-HDHA	0.18
11	Eicosapentaenoic acid	5-HEPE	0.16
12		8-HEPE	0.17
13		9-HEPE	0.36
14		11-HEPE	2.11
15		12-HEPE	0.18
16		15-HEPE	0.29
17		18-HEPE	0.21
18	Arachidonic acid	5-HETE	0.16
19		8-HETE	1.97
20		9-HETE	1.88
21		11-HETE	0.20
22		12-HETE	0.27
23		15-HETE	0.20
24	Linoleic acid	9-HODE	0.24
25		13-HODE	0.12
26	Linolenic acid	9-HOTrE	0.34
27		13-HOTrE	0.34
28	Dihomo linolenic acid	8-HeTrE	0.37
29		12-HETrE	0.20
30		15-HeTrE	0.20

Table S2. Detection limits for the complex oxylipins analyzed in this study.

No.	Metabolites	Detection limit (ng)
1	NPD-1	0.14
2	Maresin-1	0.63
3	Maresin-2	0.12
4	RvD1	0.43
5	RvD1 17(R)	0.56
6	RvD2	0.63
7	RvD3	0.55
8	RvD4 17(R,S)	0.41
9	RvD5	0.28
10	LTB4	0.11
11	LTB4 18-COOH dinor	0.37
12	LTB3	0.12
13	PGJ2 15-deoxy-delta 12,14	n.d.
14	PGB-3	n.d.
15	PGB-2	n.d.
16	LTB4 12-oxo	0.10
17	LTB5 (EPA)	0.12
18	PGJ2	n.d.
19	PGJ2 delta12	n.d.
20	LXA5 (EPA)	0.15
21	LXA4 15(R)epi	0.11
22	LXA4 6(S)	0.12
23	LXA4	0.26
24	LXB4	0.13

Table S3. Comparison of colonic concentrations of different HETE isomers in *Alox15b*-KI mice and outbred wildtype controls during the time course of DSS-induced colitis. Experimental colitis was induced in *Alox15b*-KI mice and in outbred wildtype controls as described in Materials and Methods. At different time points animals were sacrificed, colon was prepared, total lipids were extracted, extracts were hydrolyzed and the resulting free arachidonic acid oxygenation products were analyzed by LC-MS (see Materials and Methods). Metabolite concentrations were plotted in Figure 4 and the experimental raw data were evaluated using the Mann-Whitney U-test. The p-values for the pairwise comparisons are listed. Significant alterations ($p<0.05$) are labeled in green. The direction of alterations is concluded from the differences of the mean values.

metabolite	direction of alteration and p values (wildtype mice)				Direction of alteration and p values (*Alox15b*-KI mice)			
	no DSS vs. 5 d DSS	increase vs. decrease	5 d DSS vs. 8d after DSS	increase vs. decrease	no DSS vs. 5 d DSS	increase vs. decrease	5 d DSS vs. 10 d after DSS removal	increase vs. decrease
**5-HETE**	0.0001	increase	0.0048	decrease	0.0020	increase	0.2912	decrease
**8-HETE**	0.2489	increase	0.3277	decrease	0.1288	increase	0.7341	decrease
**9-HETE**	0.0059	increase	0.0360	decrease	0.0031	increase	0.6330	decrease
**11-HETE**	0.3040	increase	0.1810	decrease	0.2424	increase	0.7341	increase
**12-HETE**	0.0002	increase	0.0008	decrease	0.0054	increase	0.0176	decrease
**15-HETE**	0.0050	increase	0.0120	decrease	0.0142	increase	0.8396	decrease

Table S4. Comparison of colonic concentrations of different HEPE isomers in *Alox15b*-KI mice and outbred wildtype controls during the time course of DSS-induced colitis**.** Experimental colitis was induced in Alox15b-KI mice and in outbred wildtype controls as described in Materials and Methods. At different time points animals were sacrificed, colon was prepared, total lipids were extracted, extracts were hydrolyzed and the resulting free arachidonic acid oxygenation products were analyzed by LC-MS (see Materials and Methods). Metabolite concentrations were plotted in **Figure 5** and the experimental raw data were evaluated using the Mann-Whitney U-test. The p-values for the pairwise comparisons are listed. Significant alterations (p<0.05) are labeled in green. The direction of alterations is concluded from the differences of the mean values.

**metabolite**	**direction of alteration and p values (wildtype mice)**				**Direction of alteration and p values (*Alox15b*-KI mice)**			
	**no DSS vs. 5 d DSS**	**increase vs. decrease**	**5 d DSS vs. 10 d after DSS removal**	**increase vs. decrease**	**no DSS vs. 5 d DSS**	**increase vs. decrease**	**5 d DSS vs. 10 d after DSS removal**	**increase vs. decrease**
**5-HEPE**	0.6168	decrease	0.5287	increase	0.0119	decrease	0.0119	increase
**8-HEPE**	0.5007	decrease	0.9003	decrease	0.3493	decrease	0.1429	increase
**9-HEPE**	0.0034	decrease	0.4380	increase	0.1486	decrease	0.1363	increase
**11-HEPE**	0.9027	increase	0.0653	decrease	0.7748	increase	0.6593	increase
**12-HEPE**	0.5890	increase	0.0027	decrease	0.4540	increase	0.2945	decrease
**14-HEPE**	not quantified							
**15-HEPE**	<0.0001	increase	0.0028	decrease	0.0237	increase	0.2330	decrease
**18-HEPE**	0.9379	decrease	0.7099	decrease	0.9044	increase	0.5143	increase

Table S5. Comparison of colonic concentrations of different HDHA isomers in *Alox15b*-KI mice and outbred wildtype controls during the time course of DSS-induced colitis. Experimental colitis was induced in *Alox15b*-KI mice and in outbred wildtype controls as described in Materials and Methods. At different time points animals were sacrificed, colon was prepared, total lipids were extracted, extracts were hydrolyzed and the resulting free arachidonic acid oxygenation products were analyzed by LC-MS (see Materials and Methods). Metabolite concentrations were plotted in **Figure 6** and the experimental raw data were evaluated using the Mann-Whitney U-test. The p-values for the pairwise comparisons are listed. Significant alterations (p<0.05) are labeled in green. The direction of alterations is concluded from the differences of the mean values.

metabolite	direction of alteration and p values (wildtype mice)				Direction of alteration and p values (*Alox15b*-KI mice)			
	no DSS vs. 5 d DSS	increase vs. decrease	5 d DSS vs. 10 d after DSS removal	increase vs. decrease	no DSS vs. 5 d DSS	increase vs. decrease	5 d DSS vs. 10 d after DSS	increase vs. decrease
**4-HDHA**	0.0315	increase	0.0879	decrease	0.0005	increase	0.3648	decrease
**7-HDHA**	<0.0001	increase	0.0028	decrease	<0.0001	increase	0.0879	decrease
**8-HDHA**	0.1399	increase	0.3884	decrease	0.0026	increase	0.6330	decrease
**8-HDH109**	0.0125	increase	0.2843	decrease	0.4203	decrease	0.6401	decrease
**11-HDHA**	0.0144	increase	0.0244	decrease	0.0012	increase	0.2220	decrease
**13-HDHA**	0.0010	increase	0.0010	decrease	0.0011	increase	0.1363	decrease
**14-HDHA**	<0.0001	increase	0.0008	decrease	<0.0001	increase	0.0484	decrease
**16-HDHA**	0.0002	increase	0.0120	decrease	0.0007	increase	0.6484	decrease
**17-HDHA**	<0.0001	increase	0.0016	decrease	0.0001	increase	0.1011	decrease
**20-HDHA**	<0.0001	increase	0.0028	decrease	<0.0001	increase	0.1802	decrease

Table S6. Comparison of colonic concentrations of complex oxylipins in *Alox15b*-KI mice and outbred wildtype controls during the time course of DSS-induced colitis. Experimental colitis was induced in *Alox15b*-KI mice and in outbred wildtype controls as described in Materials and Methods. At different time points animals were sacrificed, colon was prepared, total lipids were extracted, extracts were hydrolyzed and the resulting free arachidonic acid oxygenation products were analyzed by LC-MS (see Materials and Methods). Metabolite concentrations were plotted in **Figure 6** and the experimental raw data were evaluated using the Mann-Whitney U-test. The p-values for the pairwise comparisons are listed. Significant alterations ($p<0.05$) are labeled in green. The direction of alterations is concluded from the differences of the mean values.

**Metabolite**	**Direction of alteration and p value (wildtype mice)**				**Direction of alteration and p-value (*Alox15b*-KI mice)**			
	**no DSS vs. 5 d DSS**	**Increase vs. decrease**	**5 d DSS vs. 10 d after DSS**	**increase vs. decrease**	**no DSS vs. 5 d DSS**	**increase vs. decrease**	**5 d DSS vs. 10 d after DSS removal**	**increase vs. decrease**
LTB4	<0.0001	increase	0.0004	decrease	<0.0001	Increase	<0.0001	decrease
LTB4 18-COOH-dinor	0.1009	increase	0.0164	decrease	0.0006	increase	0.0791	decrease
LTB3	0.0855	increase	0.0034	decrease	<0.0001	increase	0.0066	decrease
NPD-1	<0.0001	increase	0.0010	decrease	<0.0001	increase	0.0220	decrease
Maresin-2	<0.0001	increase	0.0002	decrease	0.0051	increase	0.0462	decrease
RVD5	<0.0001	increase	0.0028	decrease	<0.0001	increase	0.0440	decrease
PGB-2	0.1373	increase	0.0663	decrease	0.0484	increase	0.8396	same
PGB-3	0.0706	decrease	0.9770	decrease	0.0832	decrease	0.1297	increase
PGJ2 15-deoxy-delta 12,14	0.0002	increase	0.0056	decrease	<0.0001	increase	0.0681	decrease

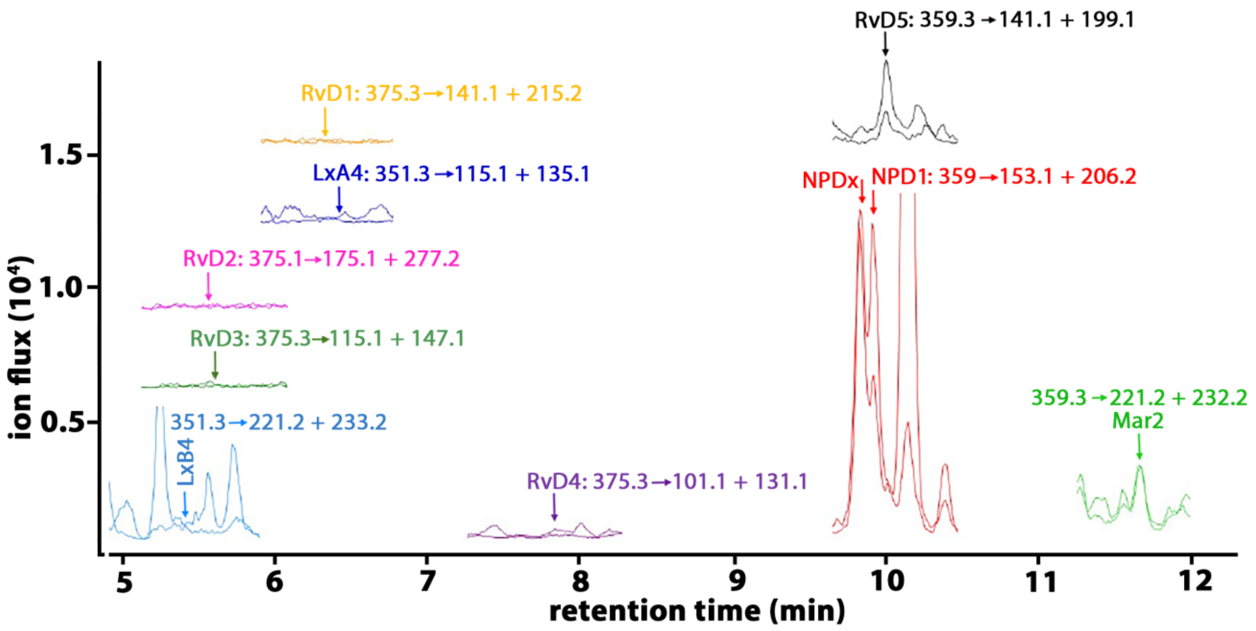

Figure S1. Partial LC-MS chromatograms for selected SPMs**:** Total lipids were extracted from inflamed colon tissues of wildtype mice and hydrolyzed under mild alkaline conditions. The hydrolyzed lipid extracts were analyzed by LC-MS as described in materials and Methods. Partial chromatograms of the regions in which authentic standards of the indicated metabolites migrated are shown. Chromatograms were followed at the expected molecular mass ion and two different fragmentation ions characteristic for the different metabolites. For RvD1, RvD2, RvD3, RvD4, LxA4 and LxB4 we did not observe significant amounts of metabolites. In contrast, for RvD5, NPDx, NPD1 and Mar2 quantifiable peaks were observed. The molecular mass ions and the fragment ions followed during chromatography are indicated for the different metabolites.
